# Effectiveness of the EIT-4-BPSD Intervention on Resident and Setting Outcomes

**DOI:** 10.1093/geroni/igab046.590

**Published:** 2021-12-17

**Authors:** Elizabeth Galik

**Affiliations:** University of Maryland School of Nursing, Baltimore, Maryland, United States

## Abstract

The effectiveness of EIT-4-BPSD was based on testing the following hypotheses: (1) Settings exposed to EIT-4-BPSD will demonstrate improvements in Environment and Policy assessments, better quality of care interactions, and more person-centered care approaches for management of behavioral symptoms in care plans compared to Education Only settings; and (2) Residents in EIT-4-BPSD settings will have fewer behavioral symptoms and less pain, maintain or improve function, use fewer psychotropic medications, and have improved quality of life compared to residents in Education Only settings. There was not a significant treatment effect at the setting or resident level. Reasons for lack of effectiveness include limited evidence of behavioral symptoms at baseline, nationally based environment and policy requirements related to behavioral management, and measurement challenges in identifying behaviors and other outcomes. Future work should focus more on process and changing how staff approach care which was demonstrated in this trial.

